# Diagnostic et traitement de la Maladie du charbon à localisation palpébrale: à propos d'un cas et revue de littérature

**DOI:** 10.11604/pamj.2013.15.94.2720

**Published:** 2013-07-11

**Authors:** Zouheir Hafidi, Hanan Handor, Mina Laghmari, Najat Handor, Lalla Ouafae Cherkaoui, Samira Tachfouti, Myriame Seffar, Rajae Daoudi

**Affiliations:** 1 Université Mohammed V Souissi, service d'ophtalmologie A de l'hôpital des spécialités, Centre hospitalier universitaire, Rabat, Maroc; 2 Université Mohammed V Souissi, Laboratoire de bactériologie de l'hôpital des spécialités, Centre hospitalier universitaire, Rabat, Maroc

**Keywords:** Maladie du charbon, oeil, œdème, blépharoplastie, Anthrax, eye, edema, blepharoplasty

## Abstract

L′anthrax est une zoonose causée par le *Bacillus anthracis*. les humains contractent généralement cette maladie dans des régions endémiques, par contact direct avec des animaux infectés ou avec leurs produits contaminés. Les localisations palpébrales sont rares dans la pratique clinique et posent des problèmes de diagnostic différentiel. Les auteurs rapportent l'observation d'un patient admis dans un tableau de cellulite préseptale, avec escarre noirâtre étendue de la paupière supérieure et œdème extensif de l′hémiface, faisant suspecter une localisation palpébrale de la maladie du charbon. L'examen bactériologique a permis de confirmer le diagnostic. Le patient a bénéficié d′une antibiothérapie à base de pénicilline G avec une bonne évolution.

## Introduction

La maladie du charbon est une anthropozoonose causée par un bacille gram positif nommé Bacillus anthracis. Cette affection peut atteindre les êtres humains lors d'une exposition à des animaux infectés, leurs tissus ou leurs produits, ou dans le cadre d'un bioterrorisme ce qui a suscité un intérêt croissant de cette affection auprès de l'opinion internationale en général et les professionnels de santé en particulier [1]. Bien que l'atteinte cutanée constitue la forme la plus fréquente, les localisations palpébrales restent très rares et posent des problèmes de diagnostic différentiel. Le diagnostic de certitude repose sur l'examen bactériologique. Mais il peut être évoqué devant un tableau d'œdème facial avec escarre noire. Le traitement à base de pénicilline G permet une guérison complète et évite les complications de cette maladie grave. La prévention passe essentiellement par la vaccination des troupeaux. Nous rapportons l'observation d'un cas d'anthrax à localisation palpébrale.

## Patient et observation

Il s'agit d'un patient âgé de 25 ans, fermier de profession habitant la région de Sidi Kacem, connue comme zone d'endémie de la maladie du charbon, admis aux urgences dans un tableau de cellulite préseptale de l'orbite gauche.

A l'interrogatoire il n'y avait pas de cas similaires dans l'entourage mais il y avait un cas de mort suspecte au sein du bétail bétail. Le début de la symptomatologie remontait à vingt-quatre jours avant l'admission par l'apparition d'une papule au niveau de la paupière supérieure gauche suite à la piqure d'un insecte non identifié, puis apparition d'un prurit et de signes inflammatoires à type de rougeur et d'œdème palpébraux; l'œdème s'est ensuite progressivement étendu à la paupière inférieure, empêchant l'ouverture palpébrale, puis à l'ensemble de la région temporale et jugale; une grande vésicule a ensuite pris place en regard de l'ancienne papule qui s'est rapidement rompue laissant place à des croutes noires. Les signes généraux se sont limités à des céphalées modérées sans notion de fièvre avec un état général bien conservé.

**Figure 1:**
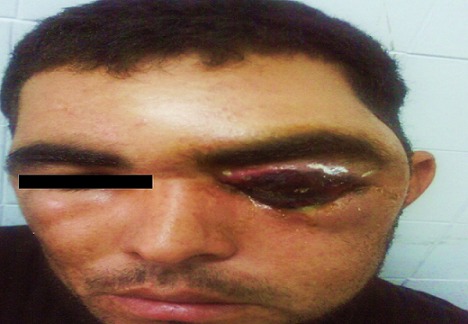
Aspect à l'admission: légère infiltration œdémateuse de la région orbitaire droite escarre noire occupant toute la paupière supérieure gauche entourée de quelques vésicules séro-sanguinolontes, associée à un œdème indolore de l'hémiface gauche

**Figure 2:**
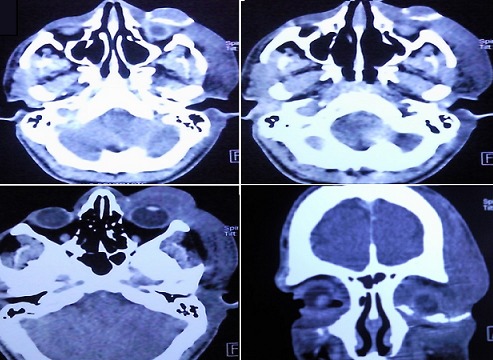
TDM orbito-cérébrale montrant une infiltration des tissus mous de l'hémiface gauche avec une calcification du plan sous cutané. Le globe oculaire semble intact

**Figure 3:**
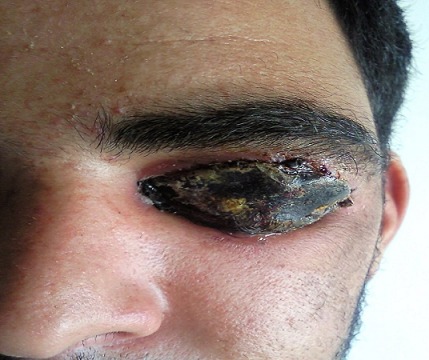
Aspect clinique 5 jours après l'instauration du traitement: régression de l'œdème avec assèchement des vésicules et transformation en escarres nécrotiques

**Figure 4:**
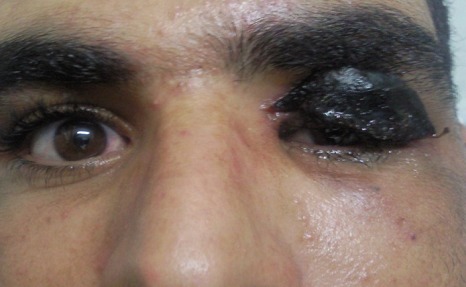
Aspect clinique 3 semaine après le traitement: nette diminution de la taille de l'escarre

**Figure 5:**
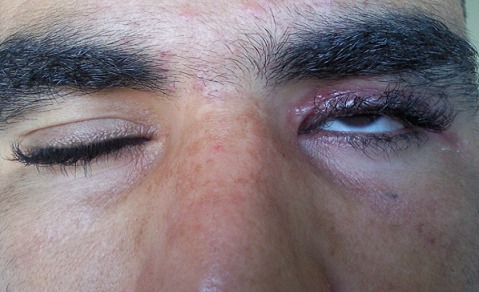
Chute de l'escarre à la fin de la 4e semaine laissant comme séquelle une lagophtalmie avec exposition cornéenne

**Figure 6:**
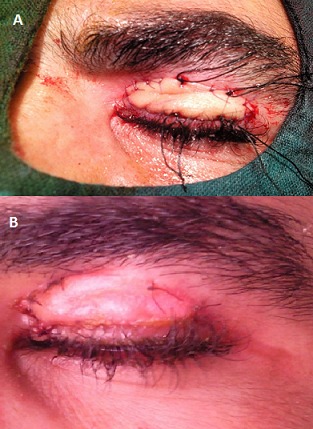
A) Aspect per opératoire de la blépharoplastie; B) Résultat de la blépharoplastie 1 semaine en post opératoire

L'examen ophtalmologique à l'admission Notait une escarre noire occupant toute la paupière supérieure gauche entourée de quelques vésicules sérosanguinolontes, associée à un œdème indolore de l'hémiface gauche empêchant toute ouverture de la fente palpébrale. On notait également une légère infiltration œdémateuse de la région orbitaire droite (Figure 1). Une TDM orbito-cérébrale a montré une infiltration des tissus mous de l'hémiface gauche avec une calcification du plan sous cutané. Le globe oculaire était sans anomalies ainsi que les structures intracérébrales notamment des sinus veineux libres (Figure 2). Un prélèvement pour étude cytobactériologique a été réalisé au niveau des vésicules. L'examen direct a montré des bacilles gram positifs à bouts carrés immobiles faisant évoquer un Bacillus anthracis, associés à une réaction cellulaire modérée. La culture a confirmé les résultats de l'examen direct, et l'antibiogramme a montré une sensibilité du germe aux pénicillines, fluoroquinolones, cyclines, phénicolés, aminosides, macrolides, pénèmes, rifampicine, et à la vancomycine, et une résistance aux céphalosporines, au triméthoprime et au sulfonamide. Le patient a été mis alors sous pénicilline G par voie intraveineuse, à raison de 5 millions d'unités toutes les 6 heures pendant 3 semaines. L'évolution a été marquée par une régression progressive de l'œdème à partir du 5e jour avec assèchement des vésicules et leur transformation en escarres nécrotiques (Figure 3), la taille des escarres a nettement diminuée à partir de la 3e semaine (Figure 4) de traitement avec chute de celles-ci vers la fin de la 4e semaine laissant comme séquelle une rétraction palpébrale responsable d'une lagophtalmie (Figure 5). Une blépharoplastie par lambeau rétro auriculaire a permis un allongement palpébral avec un résultat anatomique satisfaisant (Figure 6).

## Discussion

Le charbon est une anthropozoonose grave, anciennement décrite, mais qui a connu une recrudescence depuis les années 80 [2]. Elle est caractérisée par un potentiel élevé de dissémination ce qui en fait une redoutable arme biologique [3].

Certains pays sont considérés par l'OMS comme zones d'endémie à savoir l'Asie, l'Afrique, certains pays d'Europe et d'Amérique et certaines régions d'Australie [4]. Au Maroc Cette anthropozoonose sévit sous forme de petits foyers épidémiques; avec 72 cas enregistrés entre 2003 et 2007 [5].

L'agent pathogène est le *Bacillus anthracis*, bacille gram positif aéro-anaérobie, appartenant au groupe *Bacillus cereus*, germe très sensible aux antibiotiques dans sa forme végétative. Cette forme est repérable chez l'homme ou l'animal malade ou sur les cadavres récents; mais elle peut sporuler dans certaines conditions, comme l'exposition à l'air [1], et devenir très résistante à de nombreux désinfectants, la chaleur, la sècheresse et la lumière du soleil. Ainsi les spores peuvent demeurer viables pendant des dizaines d'années dans les peaux, le cuir et la laine d'animaux infectés et même dans le sol et l'air contaminés.

La transmission peut se faire selon plusieurs modes, majoritairement transcutanée, ce qui explique la prédominance des formes cutanées, qui représentent 95% des formes [6]. Les autres modes de transmission sont représentés par la voie respiratoire et digestive, certains auteurs ont soulevé la possibilité de transmission par piqure d'insectes [2].

Chez notre patient ce mécanisme a été suspecté devant la notion de piqure d'insecte clairement rapporté à l'interrogatoire, mais nous avons retenu une transmission transcutanée vu la profession à risque et la notion de mort inexpliquée de bétail les jours précédant la déclaration de la maladie.

Ces modes de transmission expliqueraient les différentes présentations cliniques que peut revêtir la maladie. L'évolution peut se faire vers une septicémie ou une méningite surtout en l'absence de traitement [6]. La localisation palpébrale reste rare, [7] et peut se présenter sous trois formes cliniques: la pustule maligne, l'œdème malin et la forme bulleuse.

La pustule maligne [8]: représente la forme la plus fréquente et se manifeste, après une incubation de 2 à 10 jours, par une papule érythémateuse indolore et prurigineuse de diamètre variable allant de 2 à 5 mm de diamètre. Un anneau de petites vésicules apparaît autour de la papule, celle-ci va augmenter de taille et s'excorier pendant les 2 jours qui suivent, aboutissant à la formation d'un ulcère à bord réguliers de 1 à 3 cm de diamètre [9]. L'évolution se fera au bout de quelques jours vers la nécrose et l'asséchement de l′ulcère entraînant la formation d'une escarre noire caractéristique fermement adhérente aux tissus sous-jacents et souvent entouré d′un œdème [10]. L'escarre tombe après 1 à 2 semaines, laissant une cicatrice rétractile qui pose des problèmes particuliers à la localisation palpébrale, notamment les troubles de la statique et la rétraction palpébrales avec lagophtalmie et risque d'exposition cornéenne. Le traitement est alors chirurgical et repose essentiellement sur une greffe tégumentaire [11]. Chez notre patient la chute de l'escarre au bout de la 3^e^ semaine a laissé place à une rétraction palpébrale supérieure entrainant une lagophtalmie avec exposition cornéenne ce qui a nécessité une blépharoplastie.

Bien que le globe oculaire ne soit généralement pas touché, des cas de phlébite de l′orbite, le sinus ou de la dure-mère ont été rapportés [8]. Ainsi la TDM cérébro-orbitaire et l'examen ORL ont été jugés nécessaires chez notre patient notamment devant la notion de céphalées.

La deuxième manifestation clinique est l′œdème malin des paupières qui se distingue de la pustule maligne par l′absence de l'escarre centrale et la présence d′œdème majeur [9]. Il peut être très vaste et évoluer sur un mode septicémique, qui peut conduire à des complications potentiellement mortelles, tel que le collapsus cardiovasculaire [8].

La forme bulleuse du charbon cutané n′est pas bien élucidée dans la littérature. Elle commence habituellement par un petit groupe de vésicules ou bulles des paupières. Devenant par la suite hémorragique et nécrotique, avec possibilité d'extension à l′ensemble du visage [12]. Notre patient rentre dans le cadre de la pustule maligne avec apparition d'une escarre noire entourée d'un important œdème qui est très évocateur de la maladie du charbon [10]. Bien que ces différents aspects cliniques soient évocateurs de la pustule maligne ou de l'œdème malin, et permettent souvent de soulever le diagnostic de la maladie du charbon palpébral; il ne faut pas écarter la possibilité d'autres infections d'inoculation responsables d'ulcérations avec escarre (Table 1) [13], ce qui impose le recours à l'argument bactériologique qui repose sur la découverte de l'agent (*Bacillus anthracis*) dans la pustule. En cas de négativité de l'examen bactériologique, il existe une panoplie d'autres tests qui permettent de conforter le diagnostic tel que le test des plasmides pXO1, pXO2 et du chromosome DNA [14]. Chez notre patient la positivité de l'examen bactériologique a permis de confirmer le diagnostic et de démarrer un traitement antibiotique à base de pénicilline G à raison de 5 millions d'UI par jours amenant à une rémission complète. En effet un test thérapeutique positif constitue un argument de plus surtout en cas de négativité des prélèvements à destinée bactériologique [8]. Le traitement à base de pénicilline G à raison de 5 à 20 millions d'unité par jour pendant 7 à 10 jours constitue le traitement de référence [13]. Cependant, depuis l'isolement de souches productrices de bêtalactamases résistant à la pénicilline, le Center for Disease Control (CDC) recommande en première intention la ciprofloxacine ou la doxycycline per os dans les formes cutanées bénignes et parentérales dans les formes cutanées sévères [15].

**Table 1:** Principaux diagnostics différentiels de la maladie du charbon

Lésion débutante	Piqûre d'insecte
Escarre ou ulceration	Aspergillose
	Ecthyma gangrenosum
	Ecthyma staphylococcique ou streptococcique
	Fièvre après morsure de rat (infections à Streptobacillus moniliformis, Spirillum minus)
	Fièvre vésiculeuse (rickettsiose varicelliforme)
	Leishmaniose cutanée
	Lèpre
	Morsure d'araignée
	Morve
	Mucormycose
	Nécrose à la coumadine
	Nécrose à l'héparine
	Nodules des trayeurs
	Peste
	Rickettsioses
	Streptobacillose
	Tuberculose cutanée
	Tularémie
	Typhus des broussailles, fièvres boutonneuses (rickettisoses)

Un vaccin humain acellulaire est actuellement disponible aux États-Unis (souche atténuée de *B. anthracis*) et au Royaume-Uni. Et s'adresse aux personnes à haut risque d'exposition en pré exposition (personnel de laboratoire potentiellement en contact avec les spores de *B. anthracis*, ouvriers de tanneries et d'autres lieux de traitement des peaux, de laines et d'os d'animaux dans lesquels les mesures de contrôle ne sont pas suffisantes, vétérinaires exerçant dans des zones à haute incidence de maladie du charbon animal et forces militaires intervenant dans les menaces bioterroristes), et en cas d'exposition en association à la chimioprophylaxie [15].

## Conclusion

La localisation palpébrale de la maladie du charbon est rare et doit être suspectée chez tout patient présentant une escarre noire avec œdème péri lésionnel surtout dans un contexte évocateur (sujets à risque). La confirmation diagnostique est apportée par l'isolement du Bacillus anthracis à l'examen bactériologique d'un prélèvement réalisé au niveau de l'ulcère. Le traitement curatif repose sur une antibiothérapie adaptée amenant souvent à une guérison complète. Il s'agit d'une pathologie à déclaration obligatoire et seules des mesures préventives permettront de limiter les répercutions sanitaires et économiques de cette maladie à haut potentiel de dissémination.
